# Innovative Approaches to Healthcare Access, Communication, and Culturally Respectful Care for LGBTQ+ Older Adults

**DOI:** 10.1093/geroni/igaf122.1811

**Published:** 2025-12-31

**Authors:** Korijna Valenti, Jennifer Carnahan

## Abstract

LGBTQ+ aging research increasingly underscores the necessity of inclusivity, targeted provider education, and systemic responsiveness in healthcare settings. This symposium integrates findings from four complementary studies addressing these crucial areas among older LGBTQ+ populations. Dr. Valenti and colleagues apply Minority Stress Theory and Queer Gerontology frameworks to highlight affirming healthcare experiences of older gay men living with serious illnesses in the Deep South. Their analysis identifies clinician-patient connection, respectful communication, and sexuality disclosure as key facilitators of genuinely patient-centered care. Dr. Bybee presents an innovative educational method employing Advance Directive Mini-Plays, demonstrating that interactive theater significantly enhances healthcare providers’ understanding and confidence in facilitating advance care planning discussions with LGBTQ+ patients and their support networks. Dr. Lee and colleagues offer results from a nationwide landscape assessment of the VA’s Home-Based Primary Care program, identifying substantial gaps in staff training and preparedness to support older sexual and gender minority veterans effectively. Although clinicians expressed general awareness of unique LGBTQ+ healthcare needs, findings underscore a critical demand for enhanced, tailored training resources to promote affirming care practices. Dr. Hilgeman’s formative evaluation utilizing the Long-Term Care Equality Index in a Veterans Community Living Center provides measurable evidence of improvements in inclusivity across resident services, employee benefits, and community engagement through structured assessments, comprehensive staff training, and targeted policy enhancements. Collectively, these presentations highlight advancements achieved, barriers remaining, and strategies required to systematically ensure equitable and affirming healthcare experiences for LGBTQ+ older adults, offering practical implications for future research, policy, and clinical practice improvements. Rainbow Research Group Interest Group Sponsored Symposium

